# Effects of Exercise Intervention on Health-Related Quality of Life in Patients with Systemic Lupus Erythematosus: A Systematic Review and Meta-Analysis of Controlled Trials

**DOI:** 10.3390/healthcare9091215

**Published:** 2021-09-15

**Authors:** Ming-Chi Lu, Malcolm Koo

**Affiliations:** 1 Division of Allergy, Immunology and Rheumatology, Dalin Tzu Chi Hospital, Buddhist Tzu Chi Medical Foundation, Dalin, Chiayi 62247, Taiwan; e360187@yahoo.com.tw; 2 School of Medicine, Tzu Chi University, Hualien City 97004, Taiwan; 3 Graduate Institute of Long-Term Care, Tzu Chi University of Science and Technology, Hualien City 970302, Taiwan; 4 Dalla Lana School of Public Health, University of Toronto, Toronto, ON M5T 3M7, Canada

**Keywords:** exercise, physical activity, quality of life, systemic lupus erythematosus, systematic review, meta-analysis

## Abstract

Exercise and physical activity have been deemed as potentially beneficial for patients with systemic lupus erythematosus (SLE). This study aimed to evaluate the effects of exercise interventions on health-related quality of life in patients with SLE using a systematic review and meta-analysis. Randomized and non-randomized controlled trials published up to July 2021 were examined using the PubMed and Embase databases. Of the 1158 articles retrieved, nine were included for systematic review. Five of them were randomized controlled trials and these were assessed using meta-analysis. Hedges’ *g* effect size was 0.47; 95% (confidence interval 0.21–0.73; *p* < 0.001) for the physical health and function aspect of health-related quality of life. None of the other seven domains of the SF-36 showed a significant effect size. However, the latter finding was limited by the small number of available trials. In conclusion, this systematic review and meta-analysis supported that exercise intervention compared to usual care might be able to improve the physical functioning domain of health-related quality of life in patients with SLE. Future high-quality randomized controlled trials that incorporate disease-specific health-related quality of life measures are needed to elucidate the role of exercise on health-related quality of life in patients with SLE.

## 1. Introduction

Systemic lupus erythematosus (SLE) is a chronic inflammatory autoimmune disorder with diverse disease manifestations. Women between puberty and menopause are typically predisposed to developing the condition. The reported worldwide incidence and prevalence of SLE vary considerably with incidence ranges from 1 to 10 per 100,000 person-years and prevalence ranges from 20 to 70 per 100,000 people [1]. Based on the health claim data from the Taiwan’s National Health Insurance Research Database (NHIRD), the overall prevalence of SLE between 2001 and 2011 was found to be 81 per 100,000 with a female to male ratio of 9.1 [2]. The treatment options available for SLE remain limited compared to those for other rheumatic diseases. The biologic belimumab, a B-lymphocyte stimulator-specific inhibitor, remains the only new therapy that received regulatory approved for the treatment SLE in the last 50 years [3].

The etiology of SLE is unknown and currently there is no cure available. Individuals with SLE can experience significant symptoms, such as pain, fatigue, arthritis, and rashes affecting many facets of their lives. In addition, immunosuppressive therapy could give rise to various long-term side effects. Not surprisingly, both the physical and psychological well-being of patients with SLE can profoundly be impacted [4,5]. The health-related quality of life (HRQOL), which can be defined as the “functional effect of disease and its treatment, as perceived (experienced) by the patient” [6], might be reduced [7,8]. Generally, the quality of life in patients with SLE is comparable with that in patients with other chronic diseases, which are lower than in the general population [9,10]. With improved survival due to the advance in SLE therapy, quality of life has increasingly become more important from patients’ perspective [11].

As the quality of life among patients with SLE not fully associated with SLE disease activity, pharmacologic treatments that are effective for SLE disease activity might not be able to improve quality of life in these patients [12]. Therefore, the benefit of non-pharmacological therapies on quality of life has been explored. A systematic review of non-pharmacologic therapies for SLE showed that 7 out of 11 studies (three aerobic exercise and four psychological interventions) indicated improvement in at least one subscale of quality of life as measured by SF-36, compared to the control [13]. Another recent systematic review of 21 randomized controlled trials and two quasi-experimental studies in patients with SLE concluded that non-pharmacological interventions as an adjunct to usual medical care might be beneficial for improving patients’ quality of life and psychological outcomes. The interventions included two diet-based interventions, four psychological interventions, six physical activity interventions, and one self-management course [14]. Nevertheless, no meta-analysis assessing the effect of exercise on quality of life in patients with SLE is yet available.

Previous meta-analyses on exercise in patients with SLE have mainly focused on the management of fatigue and disease activity. A meta-analysis of two randomized controlled trials and one quasi-experimental study showed that aerobic exercise could reduce fatigue and increase vitality for patients with SLE [15]. Another meta-analysis of 11 studies with 469 participants with SLE concluded that therapeutic exercise programs could improve fatigue, depression, and physical fitness without a deleterious effect on disease activity [16]. Moreover, a meta-analysis of 15 randomized controlled trials involving 846 participants with SLE evaluated the effects of non-pharmacologic therapies, including eight which used exercise interventions. Exercise appeared to be beneficial in improving fatigue, anxiety, and depression, but not in reducing SLE disease activity. [13]. To the best of our knowledge, the evidence of exercise on quality of life had only been summarized in systematic reviews, but not in meta-analyses, of non-pharmacological interventions in patients of SLE [13,14,16]. Therefore, this study aimed to specifically assess the effect of exercise intervention on quality of life in individuals with SLE using meta-analysis.

## 2. Materials and Methods

### 2.1. Search Strategy and Study Selection

A systematic review and meta-analysis was conducted in accordance with the preferred reporting items for systematic reviews and meta-analyses (PRISMA) statement guideline [17]. A comprehensive and systematic search was performed using PubMed and Embase with search from inception through August 2021 for original articles in peer reviewed journals. Embase was used a supplement to PubMed as suggested by the Cochrane Handbook for Systematic Reviews of Interventions [18].

This study applied the population, intervention, comparison, and outcome (PICO) search strategy. Using the PICO question, population (P) = in patients with SLE; intervention (I) = exercise; comparator (C) = usual care; outcome (O) = quality of life. The following word search terms, as both keywords and controlled vocabulary, in various combinations were used: “systemic lupus erythematosus”, “SLE”, “exercise”, “physical activity”, “quality of life”, and “QOL”. All non-human and non-English studies were excluded. In addition, controlled trials, with or without random group allocation, were included.

The retrieved articles were imported into Endnote X7 (Thomson Reuters, Carlsbad, CA, USA) to remove duplications. One author (MK) screened all articles based on titles and abstracts, and the other author (MCL) checked the assessment. The full-text version of potentially eligible articles was assessed if they fulfilled the eligibility criteria. In addition to the two databases, the reference lists of included articles were scanned for additional studies that met the inclusion criteria.

### 2.2. Assessment of Risk of Bias in Included Randomized Controlled Trials

The risk of bias of the included randomized controlled trials using the Revised Cochrane Risk-of-Bias Tool for Randomized Trials (RoB 2). The following domains of bias were reported for each study: (1) bias arising from the randomization process (selection bias), (2) bias due to deviations from intended interventions (performance bias), (3) bias due to missing outcome data (attrition bias), (4) bias in the measurement of the outcome (detection bias/response bias), (5) bias in the selection of the reported result (reporting bias), and (6) overall bias. Each domain was judged as “low risk of bias”, “some concerns”, or “high risk of bias” [19]. Risk-of-bias plots were created using the robvis tool [20]. The risk of bias of the studies were first assessed by one author (MK) and reviewed by the other author (MCL) and any discrepancies were resolved by consensus.

### 2.3. Statistical Analysis

The meta-analysis was performed using the Comprehensive Meta-Analysis software, Version 2.2.064 (Biostat Inc., Englewood, NJ, USA). The standardized mean difference (SMD) for each study was calculated by comparing the mean and standard deviation between intervention and control groups. Hedges’ *g* was used to adjust the effect size based on the sample size of each study. Effect sizes were interpreted as small (0.2), moderate (0.5), and large (0.8), according to Cohen’s guidelines [21].

For studies that had two intervention groups with different exercise programs, the results from both intervention groups were combined into a single group for comparison with the control group, as recommended by the Cochrane Handbook for Systematic Reviews of Interventions [22].

Heterogeneity in the results was quantified by the Higgins’s *I*^2^ statistic, which indicates the proportion of unexplained between-study heterogeneity in the meta-analysis. *I*^2^ values of 25%, 50%, and 75% were qualitatively classified as low, moderate, and substantial, respectively. The combined effect analysis of non-heterogeneous studies was conducted by the fixed effects model, whereas the random effects model was adopted for heterogeneous studies.

## 3. Results

### 3.1. Search Results

**Figure 1:**
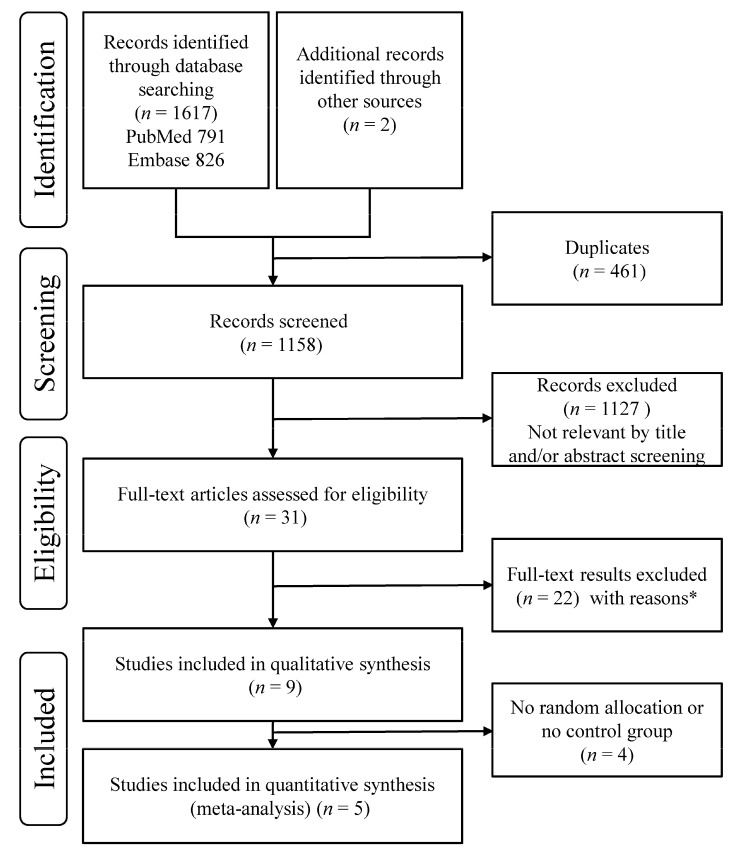
Preferred Reporting Items for Systematic Reviews and Meta-Analyses (PRISMA) flow information diagram used to select studies on the effect of exercise on quality of life in patients with systemic lupus erythematosus. * Full-text articles were excluded for the following reasons: review articles (*n* = 8), not clinical trials (*n* = 8), did not measure quality of life (*n* = 3), not in English (*n* = 1), a qualitative study (*n* = 1), and a letter (*n* = 1).

After the removal of duplicates, 1158 records were screened. Of those, 1127 records were excluded based on title and abstract. Full texts were obtained and evaluated for the remaining 31 records. Nine studies met the inclusion criteria for systematic review. Of those, four studies were excluded from the meta-analysis because either random allocation or a control group was not available (Figure 1).

### 3.2. Characteristics of Included Studies

The details of the study characteristics are displayed in Table 1. The nine included studies were published between 2000 and 2021, and were conducted in North America, South America, and Europe. The nine studies, in total, involved 458 patients with SLE (six men), with study sample sizes at the beginning ranging between 10 and 93. The intervention period in these studies ranged between six weeks and one year.

Of the nine included studies, five were randomized controlled trials and were included in the meta-analysis [23,24,25,26,27], two studies did not use random allocation in their group assignment [29,31], one study was a pilot study without the use of a usual care control group [28], and one study compared aerobic with isotonic exercise without the use of random group assignment [30].

### 3.3. Intervention Characteristics of Included Studies

Of the nine included studies, seven evaluated the effects of cardiovascular exercise, one explored the effects of whole-body vibration exercise [27], and one assessed the impact of upper limb exercise [26]. The types of exercise included walking, cycle ergometry, aerobics, and treadmill. Most of the studies incorporated some form of regular or tapering supervision even when the exercise was performed at home. For example, patients participating in a 12-week exercise program at home were asked to be seen every two weeks for a supervised exercise session [23]. Another study divided the exercise program into two phases where the exercise was supervised in the first three months but not in the subsequent six months [28]. To enhance adherence in studies of home exercise intervention, one study asked to the participants to complete an exercise diary after completion of the daily exercise program [26] and another study conducted periodical individual coaching consisted of a discussion of the patient’s motivation and barriers to exercise as well as the pros and cons of physical activity [25]. Collectively, exercise intervention length ranged from 6 to 52 weeks with 12 weeks being the most common.

### 3.4. Characteristics of the Outcome Measures

Of the nine included studies, all but one used the SF-36 to assess health-related quality of life. Five studies reported the results of all eight domains of the SF-36. One study reported only the physical function domain [28]. Another non-randomized controlled trial reported only physical fitness and vitality [29]. In the randomized controlled trial by Tench et al., only the physical function, role physical, and vitality domains were reported [23]. Moreover, one study reported only the results of the two global domains of physical and mental components of the SF-36 [31]. Furthermore, only one study used a disease-specific health-related quality of life measure, the LupusQoL [32], instead of the generic SF-36. However, the authors used only two of the eight domains of the LupusQoL, namely, physical health and fatigue, in their study [26].

### 3.5. Effects of Interventions: Exercise versus Control

In a non-randomized control trial, Gavilán-Carrera et al. reported that 12 weeks of aerobic exercise on a treadmill was not associated with a significant difference in the physical and mental global component of the SF-36 compared with the controls. In a pilot study of 10 female patients with SLE, Ramsey-Goldman et al. reported that two months of supervised aerobic exercise or range of motion/muscle strengthening exercise followed by six months of unsupervised home exercise did not lead to a significant improvement in the physical function domain of the SF-36 [31].

Conversely, Bogdanovic et al. reported that six weeks of aerobic exercise on a bicycle ergometer or isotonic exercise significantly improved all eight domains of the SF-36. There were no differences between the two forms of exercise. However, the study did not include a non-exercise control group for comparison [30]. In a non-randomized controlled trial, de Carvalho et al. showed that 12 weeks of supervised cardiovascular training was able to significantly improve two (physical fitness and vitality) of the eight domains of the SF-36 compared with the control group [29].

In the five studies based on a randomized controlled design, three showed that an exercise program could improve some domains of quality of life. In a three-arm randomized controlled trial, Abrahão et al. found that 12 weeks of cardiovascular training was able to improve the physical role functioning and vitality domains of the SF-36 [24]. Similarly, Keramiotou et al. showed that daily 30-min upper limb exercise could significantly improve the physical health and fatigue domains of the LupusQoL compared with the usual care control group [26]. On the other hand, in a three-month supervised aerobic exercise with individual coaching followed by nine months of self-managed exercise with tapering of coaching, Boström et al. reported no significant changes over time for any of the eight SF-36 domains except for mental health [25]. Moreover, Tench et al. reported that 12 weeks of home exercise consisted mainly of walking was not associated with a significant difference in the physical function, role function, and vitality domains of the SF-36 [23]. The effects of a 12-week passive whole-body vibration intervention were evaluated by Lopes-Souza et al. and no significant differences were found in any of the eight domains of the SF-36 [27]. Overall, it appears that aerobic exercise could improve some aspects of health-related quality of life.

Because a poorer health-related quality of life was found to be associated with fatigue [33], higher organ damage [8], and disease activity in patients with SLE [34] and that those experiencing more fatigue, higher organ damage, or more severe disease activity are less likely to engage in physical activity [35], a group assignment without the use of randomization would increase the risk of selection bias due to differences in group characteristics at the baseline, including those that can affect the outcome measure. Therefore, only five randomized controlled trials were included in the following meta-analysis.

### 3.6. Risk of Bias in Included Randomized Controlled Trials

**Figure 2:**
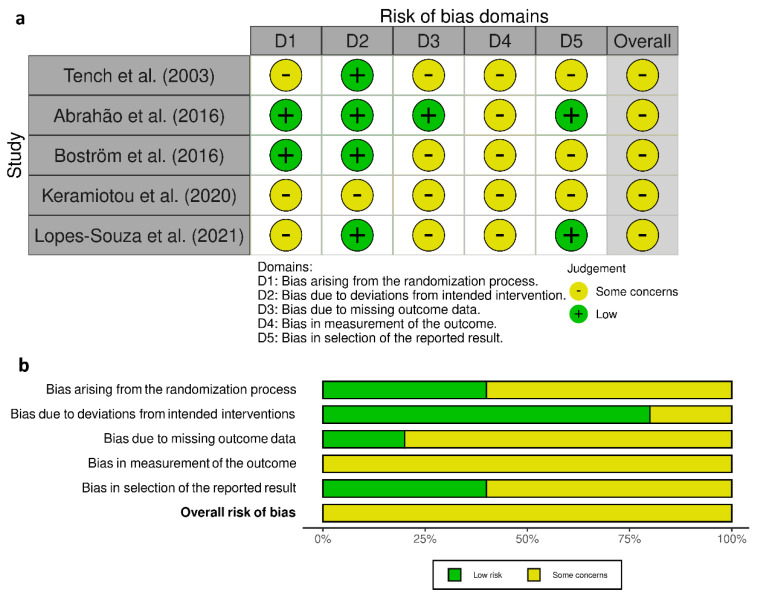
Risk of bias assessment: (**a**) Risk of bias summary. (**b**) Risk of bias graph.

Results of the assessment of risk of bias for the included randomized controlled trial are summarized in Figure 2. While all studies indicated that the participants were randomly allocated to different groups, only two of them had explicitly reported details about random sequence generation and allocation concealment [24,25]. In addition, four of the studies had some concerns for attrition bias. For example, in the study by Keramiotou et al., seven patients from the exercise group and six from the control group did not start the exercise program after they had agreed to participate [26]. Although the authors of the study mentioned that the reasons for dropout were irrelevant to the program, it could potentially be associated with quality of life measures. Furthermore, only two studies mentioned that their study protocols were registered at a local or international trial registry [24,27]. Finally, the lack of blinding of participants and personnel was the main weakness across all included studies.

### 3.7. Meta-Analysis

As not all included studies used the SF-36 health-related quality of life measure or all of its subscale, nine separate meta-analyses were conducted, including: one analysis on all five studies regardless of the health-related quality of life measure; another analysis on four studies that used the physical function domain of the SF-36; and seven analyses on studies that used the remaining seven domains of the SF-36.

**Figure 3:**
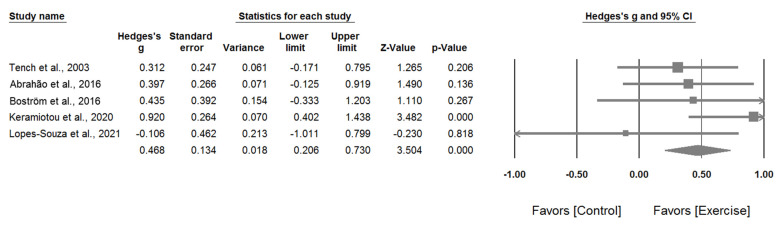
Forest plot of comparison with the outcome of the physical health and function aspect of health-related quality of life.

First, the results of the meta-analysis of the five randomized controlled trials showed a significant positive effect of exercise on the physical health and function aspect (physical function in the SF-36 and physical health in LupusQoL) of health-related quality of life among patients with SLE (Hedges’ *g*: 0.468; 95% confidence interval [CI]: 0.206–0.730; *p* < 0.001). Heterogeneity between studies was low and not significant (*I*^2^ = 19.2%; *p* = 0.292) (Figure 3).

**Figure 4:**
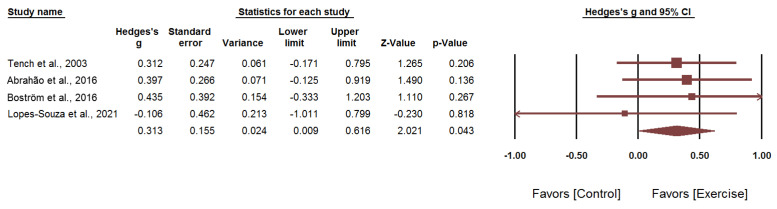
Forest plot of comparison with the outcome of the physical function domain of the SF-36.

Second, Figure 4 showed the results with the one study that used the LupusQoL removed. The pooled results of the four studies on the physical function domain of the SF-36 also showed a significant positive effect of exercise on the physical function domain of the SF-36 among patients with SLE (Hedges’ *g*: 0.313; 95% CI: 0.009–0.616; *p* = 0.043). Heterogeneity between studies was not significant (*I*^2^ = 0%; *p* = 0.796).

**Figure 5:**
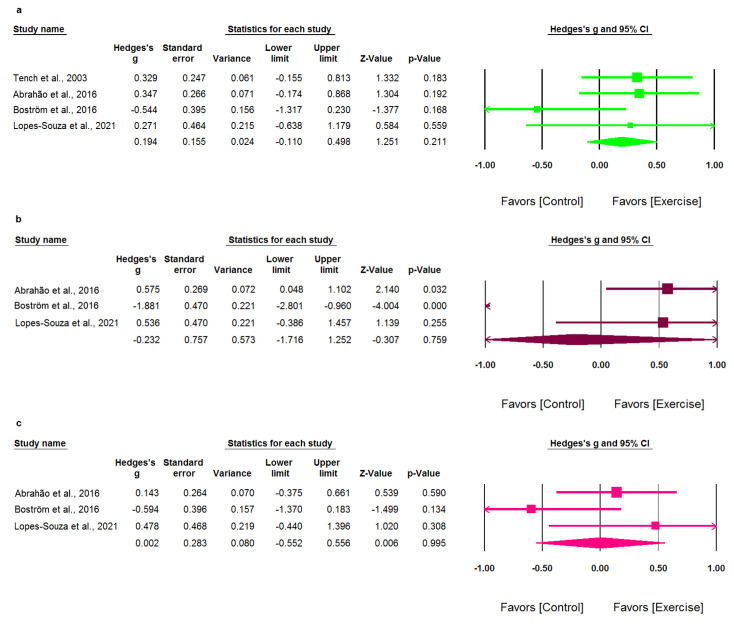

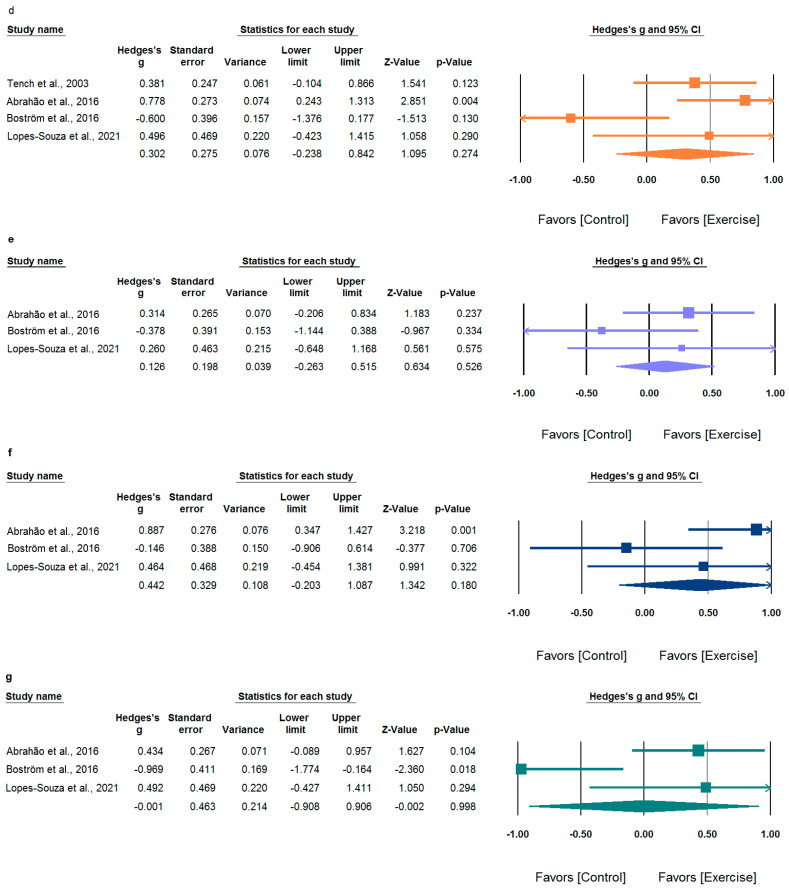
Forest plot of comparison with the outcome of the SF-36 domains other than the physical function domain: (**a**) role physical, (**b**) pain, (**c**) general health, (**d**) vitality, (**e**) social functioning, (**f**) role emotional, and (**g**) mental health.

Next, the results for the remaining seven domains of the SF-36 are shown in Figure 5a–g. Exercise showed no significant effects on all of these domains of the SF-36. The pooled results of exercise on the remaining seven domains of the SF-36 were as follows: (1) Role physical domain (Hedges’ *g*: 0.194; 95% CI: −0.110–0.498; *p* = 0.211). Heterogeneity between studies was low and not significant (*I*^2^ = 27.7%; *p* = 0.246) (Figure 5a); (2) Pain domain (Hedges’ *g*: −0.232; 95% CI: −1.716–1.252; *p* = 0.759). Heterogeneity between studies was substantial and significant (*I*^2^ = 90.8%; *p* < 0.001) (Figure 5b); (3) General health domain (Hedges’ *g*: 0.002; 95% CI: −0.552–0.556; *p* = 0.995). Heterogeneity between studies was moderate but not significant (*I*^2^ = 44.0%; *p* = 0.168) (Figure 5c); (4) Vitality domain (Hedges’ *g*: 0.302; 95% CI: −0.238–0.842; *p* = 0.274). Heterogeneity between studies was moderate and significant (*I*^2^ = 63.8%; *p* = 0.040) (Figure 5d); (5) Social functioning domain (Hedges’ *g*: 0.126; 95% CI: −0.263–0.515; *p* = 0.526). Heterogeneity between studies was low and not significant (*I*^2^ = 11.0%; *p* = 0.325) (Figure 5e); (6) Role emotional domain (Hedges’ *g*: 0.442; 95% CI: −0.203–1.087; *p* = 0.180). Heterogeneity between studies was moderate but not significant (*I*^2^ = 57.8%; *p* = 0.094) (Figure 5f); and (7) Mental health domain (Hedges’ *g*: −0.001; 95% CI: −0.908–0.906; *p* = 0.998). Heterogeneity between studies was substantial and significant (*I*^2^ = 77.9%; *p* = 0.011) (Figure 5g).

## 4. Discussion

This study investigated the effects of exercise intervention on health-related quality of life in patients with SLE. To our knowledge, this review was the first to employ meta-analysis specifically on this topic. Nine controlled trials were included in the systematic review and five of them were randomized controlled trials, which were further assessed using meta-analysis.

Nine separate meta-analyses were conducted because not all studies used the same health-related quality of life measure and all not domains of the SF-36 were assessed in each study. The physical function and health aspect of the quality of life was the only domain that covered by all five trials. We found a statistically beneficial effect favoring exercise intervention with a medium effect size of 0.468. This finding agrees with previous systematic reviews of non-pharmacological interventions, including exercise, that it could improve quality of life in patients with SLE [13,14].

A small heterogeneity (*I*^2^ = 19.2%) was observed in the meta-analysis that included five studies on the physical function and health domain of quality of life. The reason could be due to the use of the LupusQoL in one study. Indeed, when that study was excluded, the *I*^2^ value became 0%. The effect size reduced to 0.313, but was still statistically significant in favor of exercise intervention.

As not all domains of the SF-36 were measured and reported in the included randomized controlled trials, the remaining seven domains were evaluated in studies where data were available. None of the seven domains showed a significant effect size. In contrast, meta-analysis of exercise appeared to provide some benefits on quality of life in patients with other rheumatic diseases. A recent meta-analysis of 29 controlled trials in patients with rheumatic diseases (13 osteoarthritis, 9 fibromyalgia, 5 rheumatoid arthritis, 1 SLE, and 1 chronic fatigue syndrome), indicated that resistance exercise significantly improved the general health-related quality of life (effect size = 0.50), the physical role functioning (effect size = 0.41), physical functioning (effect size = 0.72), social aspects (effect size = 0.27), and body pain (effect size = 0.31) compared with control group [36]. Given the scarcity of studies on SLE, there is a need to conduct more randomized controlled trials on exercise interventions in patients with SLE.

With respect to the instruments used to measure health-related quality of life, the generic SF-36 was the most frequently used tool. Although the use of the SF-36 allows for comparison of quality of life in various diseases, it lacks the details that are characteristics of SLE, such as body image and intimate relationships [37]. SLE-specific instruments, such as the 34-item LupusQoL developed by McElhone et al. in 2007 [32], and the 40-item SLEQOL developed by Leong et al. [38], might be able to offer enhanced responsiveness to changes in health-related quality of life than the SF-36. Future studies may want to use these instruments either alone or in combination with a generic measure to ensure that both disease-specific and wider aspects of quality of life are assessed.

It is worth mentioning that in studies of exercise intervention, it is clearly difficult to blind the participant to the intervention. Therefore, bias introduced by a placebo effect can potentially overestimate the efficacy of an intervention, particularly in the evaluation of subjective outcomes. It has been estimated in studies analyzing the efficacy of different ergogenic substances on sporting performance that the variance weighted mean effect size of a placebo effect was 0.31 [39]. The mediating factors underlying the effects of exercise intervention, especially on emotional and social aspects of quality of life, will need to be further elucidated. For example, levels of social support [40] and psychological morbidity [41] could influence both health-related quality of life and these factors are known to be associated with exercise.

### Limitations

The results of the present meta-analysis should be interpreted with the following caveats in mind. First, the strength of any meta-analysis depends upon the quality of the included studies. Strong conclusions could not be drawn due to the limited quality of the included studies, particularly the absence of blinding of participants. Second, only five randomized controlled trials were available for meta-analysis in the present study. Third, none of the included studies were conducted in the Asian regions and the findings of this review might not be generalized to patients outside of the studied regions.

## 5. Conclusions

This systematic review and meta-analysis supported that exercise intervention compared to usual care might be able to improve the physical functioning domain of health-related quality of life in patients with SLE. Nevertheless, owing to the limited number of available randomized controlled trials on the topics, there was limited evidence on the positive effects of exercise intervention in other aspects of health-related quality of life. Future high-quality randomized controlled trials that incorporate disease-specific health-related quality of life measures are needed to better evaluate the possible benefits of exercise intervention on various aspects of quality of life in patients with SLE.

## Figures and Tables

**Table 1:** Summary of the included controlled trials in the systematic review and meta-analysis.

Study	Country	Sample Size at Start	Female (%)	Intervention	Control	Duration (Weeks)	QOL Scale	Main Findings Relating to QOL
Meta-analysis								
Tench et al., 2003 [23]	United Kingdom	93 IG: 33 CG1: 32 CG2: 28	100	Home exercise (walking, cycling, swimming), 30–50 min × 3 times/wk × 12 wks	CG1: Usual care CG2: Relaxation audiotape (this group was not included in the meta-analysis)	12	SF-36: 3 domains	No significant between-group differences in the physical function, role physical, and vitality domains of SF-36 after 12 wks of treatment
Abrahão et al., 2016 [24]	Brazil	63 IG1: 21 IG2: 21 CG:21	97	IG1: Cardiovascular exercise, 50 min × 3 times/wk × 12 wks IG2: Resistance exercise, 50 min × 3 times/wk × 12 wks	Usual care	12	SF-36: 8 domains	Significant improvement in quality of life from baseline to 12 wks in both exercise groups
Boström et al., 2016 [25]	Sweden	35 IG: 18 CG: 17	100	0–3 months: Supervised aerobic exercise, 60 min × 2 times/wk + education + individual coaching of physical activity + heart rate monitor + physical activity diary 4–12 months: Tapering of coaching, self-managed physical activity	Usual care	52	SF-36: 8 domains	Significant improvement in SF-36 mental health domain at 6 months
Keramiotou et al., 2020 [26]	Greece	58 IG: 28 CG: 30	94	Individually tailored 30-min daily upper-limb home exercise program 30 min daily	routine care	24	LupusQoL	Significant improvement in LupusQoL physical health and fatigue domains only in the exercise group but not in the control group
Lopes-Souza et al., 2021 [27]	Brazil	21 IG: 11 CG: 10	100	Whole-body vibration exercise, 2 times/wk × 12 wks	Isometric stance with 130° knee flexion, 2 times/wk × 12 wks	12	SF-36: 8 domains	No significant differences in any of the 8 domains of SF-36 either between wk 0 and wk 12 or between groups
Systematic review								
Ramsey-Goldman et al., 2000 [28] ^1^	USA	10 IG1: 5 IG2: 5	100	IG1: Aerobic exercise Phase1: Group exercise, 50 min × 3 times/wk × 2 months Phase2: Home exercise × 6 months IG2: Range of motion/muscle strengthening exercise Phase1: Group exercise, 50 min × 3 times/wk × 2 months Phase2: Home exercise × 6 months	–	32	SF-36: physical function domain	No significant differences in physical function of quality of life
de Carvalho et al., 2005 [29] ^2^	Brazil	60 IG: 41 CG: 19	100	Supervised cardiovascular exercise, 60 min × 3 times/wk × 12 wks	Usual care	12	SF-36: 8 domains	Significant between-group differences in the physical fitness and vitality domains of SF-36
Bogdanovic et al., 2015 [30] ^1,2^	Serbia	60 IG1: 30 IG2: 30	100	IG1: Aerobic exercise, 15 min × 3 times/wk × 6 wks IG2: Isotonic exercises, 30 min × 3 times/wk × 6 wks	–	6	SF-36: 8 domains	Significant improvement in all areas of SF-36 after aerobic or isotonic exercise, but no differences between the two types of exercise.
Gavilán-Carrera et al., 2020 [31] ^2^	Spain	58 IG: 26 CG: 32	100	Aerobic exercise on a treadmill, 75 min × 2 times/wk × 12 wks	Usual care	12	SF-36: physical health and mental health domains	No significant between-group differences in the changes in quality of life.

CG: control group; IG: intervention group; min: minutes; SF-36: Short Form 36; wk: week. ^1^ no control group; ^2^ did not use random allocation for group assignment.

## Data Availability

All data underlying the findings are within the paper.
